# Context-dependent decision-making in the primate hippocampal–prefrontal circuit

**DOI:** 10.1038/s41593-024-01839-5

**Published:** 2025-01-06

**Authors:** Thomas W. Elston, Joni D. Wallis

**Affiliations:** 1 Department of Neuroscience, University of California, Berkeley, Berkeley, CA USA; 2 Present Address: Department of Neuroscience, University of Texas at Austin, Austin, TX USA

**Keywords:** Decision, Neurophysiology, Neural circuits, Population dynamics

## Abstract

What is good in one scenario may be bad in another. Despite the ubiquity of such contextual reasoning in everyday choice, how the brain flexibly uses different valuation schemes across contexts remains unknown. We addressed this question by monitoring neural activity from the hippocampus (HPC) and orbitofrontal cortex (OFC) of two monkeys performing a state-dependent choice task. We found that HPC neurons encoded state information as it became available and then, at the time of choice, relayed this information to the OFC via theta synchronization. During choice, the OFC represented value in a state-dependent manner; many OFC neurons uniquely coded for value in only one state but not the other. This suggests a functional dissociation whereby the HPC encodes contextual information that is broadcast to the OFC via theta synchronization to select a state-appropriate value subcircuit, thereby allowing for contextual reasoning in value-based choice.

## Abstract (web summary)

The brain uses different valuation schemes across contexts. Elston and Wallis show this is supported by hippocampal encoding of context that is broadcast to prefrontal value subcircuits via theta synchronization.

## Main

Cognitive maps provide us with an internal representation of relationships, associations and structure in our environment. They serve as mental blueprints, enabling us to remember locations, plan efficient routes and generate novel plans on the fly. Although cognitive maps are famously associated with hippocampal place cells and spatial navigation^1^, recent advances point to a more generalized cognitive map theory that extends beyond spatial cognition to incorporate nonspatial domains such as value, conceptual knowledge and abstract thought^2–5^. As the concept of the cognitive map has broadened beyond just spatial information, the network of brain areas implicated in the process has also grown. Neuroimaging studies have repeatedly found that frontal areas, such as the orbitofrontal cortex (OFC) and ventromedial prefrontal cortex, are activated when subjects are required to use relational structures to solve tasks^6–8^. This has led to the theory that the primary function of the OFC is the representation of cognitive maps^9^.

Neurophysiological studies in the rodent have also started to emphasize the role that the OFC plays in constructing cognitive maps^10,11^. For example, during the learning of an odor sequence task, rats began to generalize the structure of the task across multiple problems, and this was reflected in the activity of ensembles of OFC neurons^12^. However, there are striking differences between OFC neural activity between rodents and primates, most notably in that single-neuron recordings in monkeys consistently show that the OFC typically encodes little information about spatiotemporal contingencies that would be a critical component of a map^13–17^. It may be that the primate OFC is primarily involved in using rather than constructing cognitive maps. This contrasts with the hippocampus (HPC) where neurons encode both spatial and temporal contexts, precisely the kind of information that is essential for building and initializing an internal model of the task at hand^18–20^. The HPC densely innervates the OFC^21,22^, and interactions between them via theta waves have recently been implicated in value-based choice^23^.

An as of yet untested prediction of the cognitive map theory is that value representations ought to be dependent on task states. This prediction directly contrasts with the dominant view of OFC function, which is that it encodes a ‘common currency’ that will generalize across task states^24^. To test this, we developed a state-dependent decision-making task that required subjects to make value-based decisions based on task states that varied trial by trial and recorded neural activity simultaneously in the OFC and HPC.

## Results

**Fig. 1:**
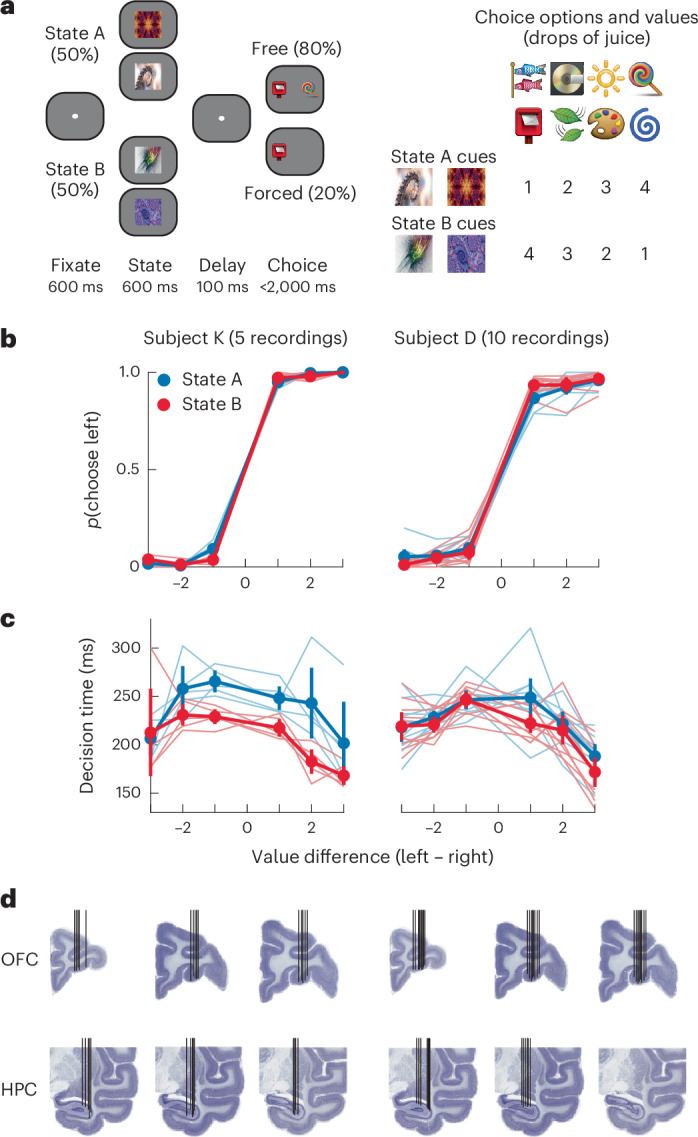
Behavioral task, performance and recording locations. **a**, Structure of the state-dependent choice task. Subjects initially fixated and were then shown one of four state cues (two per state). After a delay, subjects were presented with either one (forced choice) or two (free choice) choice options. The optimal choice depended on the state cued earlier in the trial. States were varied pseudorandomly across trials. To unconfound neuronal activity related to the physical properties of the stimuli from the meaning they signified, we used two distinct cues for each state and value. **b**, Choice as a function of the difference between option values and task state. Thin lines are single sessions, and thick lines are the mean values. The error bars represent bootstrapped 95% confidence intervals. Choice was well predicted by the difference in value and did not differ across states; *N* = 5 recordings for subject K and *N* = 10 recordings for subject D. **c**, Choice response times plotted as in **b**. Choices were slower when options were closer in value; *N* = 5 recordings for subject K and *N* = 10 recordings for subject D. **d**, Electrode trajectories and recording locations for each subject overlayed on representative Nissl-stained coronal sections. Each black vertical line indicates a single electrode trajectory. Source data

Two monkeys (subjects K and D) were trained to choose between reward-predictive pictures where the amount of reward associated with the picture depended on a task state (Fig. 1a). On each trial, subjects were cued as to whether the current trial should be evaluated according to the value scheme of state A or B. Then, after a brief delay, they were presented with either one (forced choice, 20% of trials) or two options (free choice, 80% of trials) to select from. Critically, the picture values on each trial depended on the cued task state. Therefore, to perform the task, the subjects flexibly updated the values assigned to the choice options and used these flexible state-dependent valuations to guide their responses. Both subjects were proficient at the task (Fig. 1b) and were equally accurate across both task states (subject K: state A correct trials = 98 ± 0.3%, state B = 98 ± 0.4%, paired *t*-test, *t*_4_ = 1.8, *P* > 0.1; subject D: state A = 95 ± 0.9%, state B = 93 ± 1.0%, *t*_9_ = 1.7, *P* > 0.1). We then used regression models to examine whether the animals’ decision times varied on free choice trials according to the absolute difference between offers, the side of the best offer and the task state (Methods and Fig. 1c). Both subjects responded faster when the difference between the options was larger (subject K: *β* = −5.5, *P* < 0.001; subject D: *β* = −4.9, *P* < 0.001), and we found that subject K responded faster in state B than in state A (*β* = −14, *P* = 0.002). Subject D’s decision times were unaffected by task state (*β* = −4.6, *P* = 0.07). Decision times in both subjects were not affected by the location of the best option, and there were no significant interactions (*β* < 1 and *P* > 0.1 in all cases). With respect to the forced choice trials, both subjects responded significantly faster for higher-value options and tended to respond more quickly in state A than in state B (Supplementary Fig. 1).

### Encoding of state and value information in the HPC and OFC

**Fig. 2:**
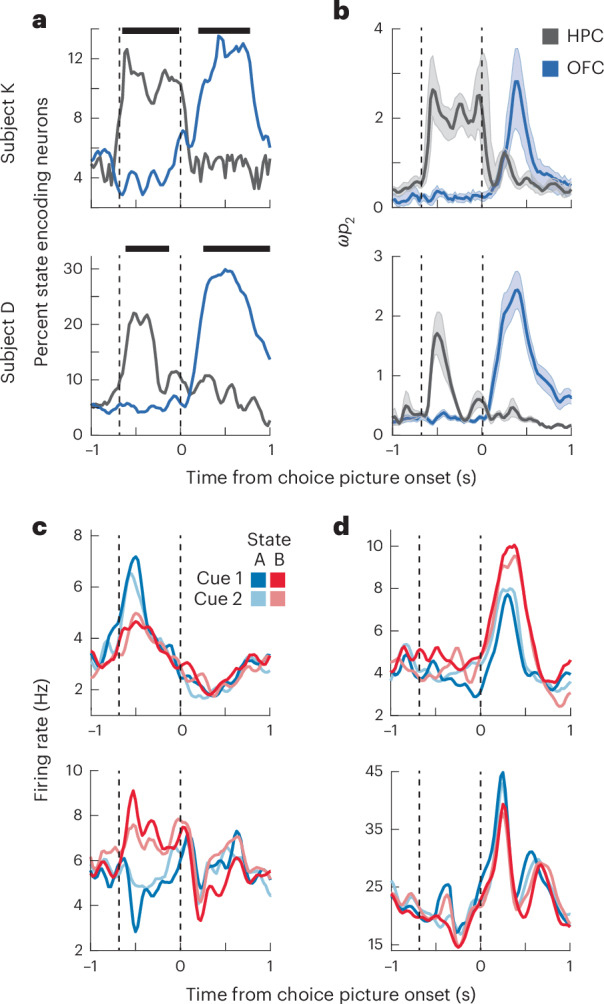
Neuronal encoding of state in the HPC and OFC. **a**, The prevalence of neurons that significantly encoded state in each brain area, as defined in a two-way ANOVA with factors of state and value. During the state epoch, HPC neurons, but not OFC neurons, encoded state information. The reverse was true in the choice epoch. Dashed vertical lines indicate the onset of the state and choice epochs, respectively. Horizontal bars indicate significant differences (two-sided *χ*^2^ test, *P* < 0.05). **b**, The strength of state encoding in the HPC and OFC as measured by *ω*_*p*_^2^, an unbiased measure of percent explained variance. Only neurons that significantly encoded state information during either the state or choice epoch are included. Dashed vertical lines indicate the onset of the state and choice epochs, respectively. The thick lines and shaded regions denote the mean and bootstrapped 95% confidence intervals, respectively. **c**, Two example HPC neurons that significantly encoded state information during the state epoch. The top neuron responded more strongly to state A, whereas the bottom neuron responded more strongly to state B. Dashed vertical lines indicate the onset of the state and choice epochs, respectively. **d**, Two example OFC neurons, plotted as in **c**, that significantly encoded state information during the choice epoch. The top neuron responded more strongly to state B, whereas the bottom neuron responded more strongly to state A. Source data

To understand the neural mechanisms of state-dependent valuation and choice, we simultaneously recorded neurons in the HPC (K = 179 neurons, D = 125 neurons) and OFC (K = 251 neurons, D = 281 neurons) as subjects performed the task (Fig. 1d). We first assessed how state tuning of individual HPC and OFC neurons evolved across the course of the trial using a sliding-window analysis of variance (ANOVA; see Methods). Across both subjects, we found that HPC neurons were more likely to encode state information than OFC neurons during the state epoch whereas the reverse was true during the choice epoch (Fig. 2). Similar results were observed during both free and forced choice trials (Supplementary Fig. 2). These results reflect a functional double dissociation of state encoding, suggesting that the HPC initially encodes state when state information becomes available, whereas the OFC encodes state information when it must be used to guide a decision.

**Fig. 3:**
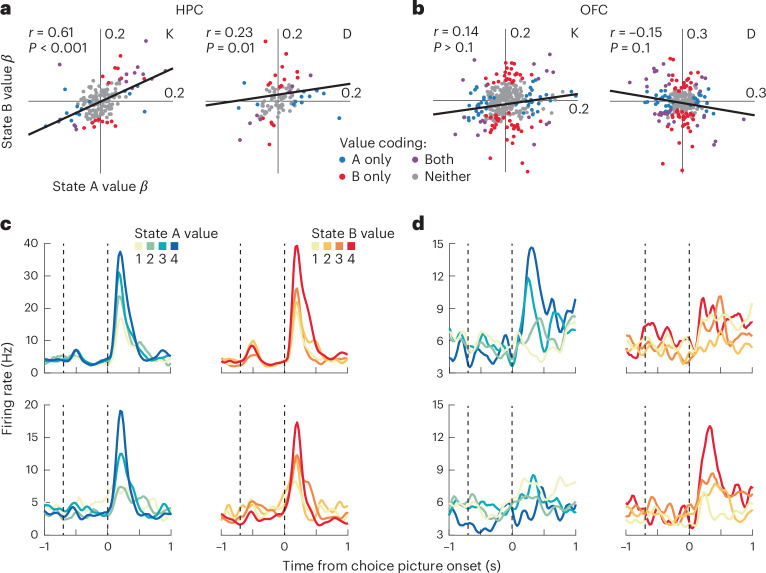
State-dependent value coding in the OFC and general value coding in the HPC. **a**, Value beta weights for each HPC neuron in state A versus state B. Each data point is a single neuron. The *r* values correspond to two-sided Pearson correlations. The exact *P* value for subject K is 2.6 × 10^−14^. **b**, Value beta weights for each OFC neuron in state A versus state B plotted as in **a**. **c**, Two example HPC neurons (top and bottom) that encoded value similarly in both task states. Dashed vertical lines indicate the onset of the state and choice epochs, respectively. **d**, Two example OFC neurons, plotted as in **c**, which encoded value in only state A (top) or state B (bottom). Source data

Next, we examined how value coding was influenced by task state. We fit a regression model to each neuron’s firing rates during the choice epoch, with predictors of state and value and their interaction (Methods). Whereas HPC neurons tended to only encode value, OFC neurons encoded the interaction between state and value (Supplementary Fig. 3). To better understand this, we quantified the value tuning of each neuron in each state with a regression coefficient (Fig. 3a,b). In both areas, many neurons significantly encoded value in at least one state (subject K: 48/179 or 27% in the HPC and 126/351 or 36% in the OFC; subject D: 54/125 or 43% in the HPC and 126/281 or 45% in the OFC). To understand whether the value coding varied by state, we correlated the resulting beta weights. If value coding was independent of state, we would expect a significant positive correlation of the value beta weights from the two regressions. Indeed, this was the case in the HPC but not the OFC (Fig. 3c,d). Furthermore, the correlation was significantly stronger in the HPC than in the OFC (*P* < 0.001 in both subjects; Fisher’s *r* to *z* transform and test). Thus, the OFC encodes state-dependent values, whereas the value coding in the HPC may be more general. We also examined whether the time course of value coding differed between the areas, but there was little evidence for any systematic difference (Supplementary Fig. 4).

**Fig. 4:**
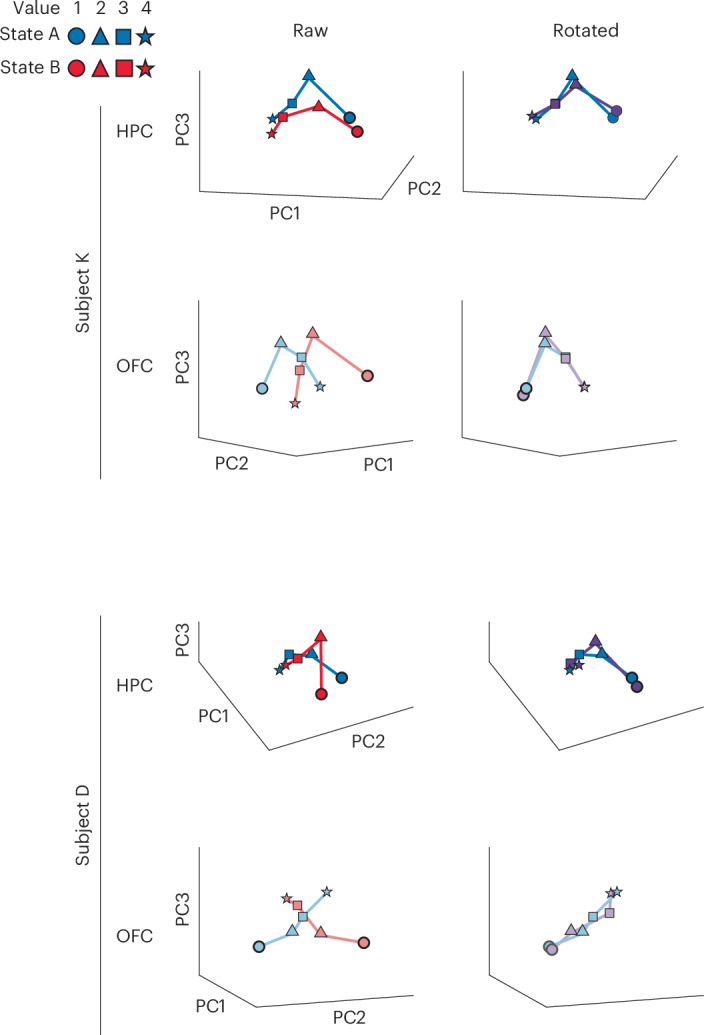
Isomorphic population value codes are orthogonal across task states in the OFC but not the HPC. Positions in PC space of each value in each task state. Each marker denotes a single value-in-state combination. Results are shown for each brain area and each subject separately. The left plots show the raw positions in PC space, whereas the right plots show the result of rotating the state B vector to align with the state A vector. Although the HPC vector requires only small rotations, the OFC vector requires almost a 90° rotation (around the vertical axis in subject K and around the depth axis in subject D). Source data

To further characterize how the value code differed between states, we examined how neuronal populations in each area varied at the ensemble level using both geometric and decoding approaches. First, we projected population activity into a low-dimensional space and geometrically assessed the similarity of value representations across task states (see Methods). In both brain areas, we found that, regardless of task state, values tended to lie along an axis, and the value hierarchy appeared to be isomorphic, that is, the overall within-state relationships between the values were similar (Fig. 4). We formally tested this via a rigid rotation analysis where the value manifold of task state B was aligned to the manifold of state A via rigid (axial) rotation. Across both brain areas and subjects, we found that this rotation was sufficient to align the value manifolds across task states, confirming that the value code is isomorphic. However, whereas the value manifold required a relatively small rotation in the HPC (mean = 34° ± 0.13°), the value manifold in the OFC was orthogonal across task states, requiring a mean 77° ± 0.15° rotation to produce maximal alignment. Consequently, the degree of rotation required to maximize cross-state manifold alignment was significantly greater in the OFC than in the HPC (bootstrapped confidence intervals had zero overlap, *P* < 0.001).

In addition, we examined how well a decoder trained on the values in one state could generalize to the alternative state. Decoding performance increases as the number of neurons in the decoder increases, so to ensure fair comparison across neuronal populations, we adopted a bootstrapping approach. In each of 1,000 bootstraps, a random set of *N* neurons was selected. We then plotted the performance of the decoders as the number of neurons increased (Supplementary Fig. 5). We found strong within-state value coding in both the OFC and HPC and strong decreases in cross-state decoding accuracy. Thus, our results across three different analyses (single-neuron tuning, rotations in principal component (PC) space and cross-context decoder generalization) all show that the value code in the OFC is state dependent.

### HPC–OFC communication via theta oscillations

**Fig. 5:**
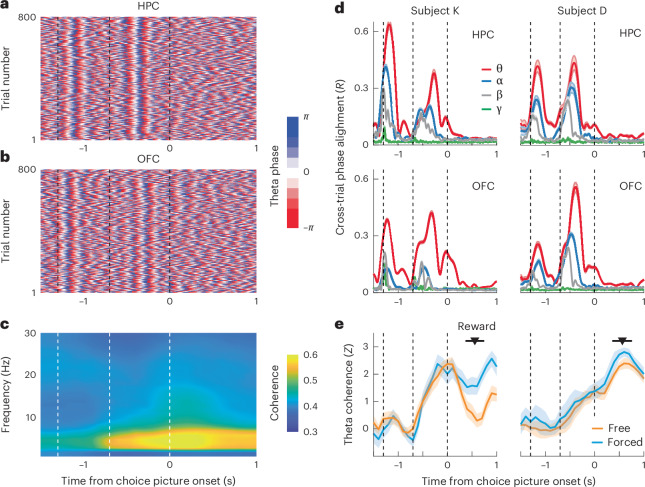
Theta activity in the HPC and OFC. **a**, HPC theta phase over the course of the trial from a representative recording session from subject K. Each row represents the instantaneous phase at each of 800 trials within a single session on a single HPC electrode. Dashed vertical lines indicate fixation, state and choice epochs, respectively. **b**, OFC theta phase, plotted as in **a**, from a representative recording session from subject K. **c**, HPC–OFC coherence from an example session from subject K. Dashed vertical lines indicate fixation, state and choice epochs, respectively. **d**, Cross-trial phase alignment at theta (4–8 Hz), alpha (9–12 Hz), beta (13–30 Hz) and gamma (30–60 Hz) frequencies. Error bars represent bootstrapped 95% confidence intervals. Dashed vertical lines indicate fixation, state and choice epochs, respectively. Theta exhibited strong phase alignment to the presentation of the state cue but not the choice options. **e**, Time course of HPC–OFC theta coherence. Theta coherence increased shortly after the onset of the state cue and peaked at the time of choice. Error bars represent bootstrapped 95% confidence intervals. Dashed vertical lines indicate fixation, state and choice epochs, respectively. *N* = 42 channel pairs for subject D, and *N* = 28 channel pairs for subject K. Source data

Our single-neuron results suggest that the HPC initially encodes state information and then relays it to the OFC so that state can inform cortical valuation and decision representations by generating state-dependent values. The HPC strongly projects to the OFC^21^, and HPC–OFC interactions in the theta band (4–8 Hz) have previously been implicated in updating OFC value representations^23^. Given that state encoding was strongest in the HPC during the cue phase but then shifted to the OFC during the choice phase, we wondered whether the structures could be communicating. First, we computed power spectrum densities to identify oscillatory activity. Across both subjects and brain areas, theta was the dominant oscillation (Supplementary Fig. 6). Theta responses were visible on nearly every individual trial and appeared to align to specific task events (Fig. 5a,b). We quantified this effect via a cross-trial phase alignment measurement that measured the mean resultant vector length, *R*, of phase angles across trials. In both subjects and both brain areas, theta phases showed strong cross-alignment at the time of fixation and at the presentation of the state cue (Fig. 5d,e). Interestingly, we did not observe a distinct response to the onset of the choice options, showing that these responses are not simply sensory-driven events.

**Fig. 6:**
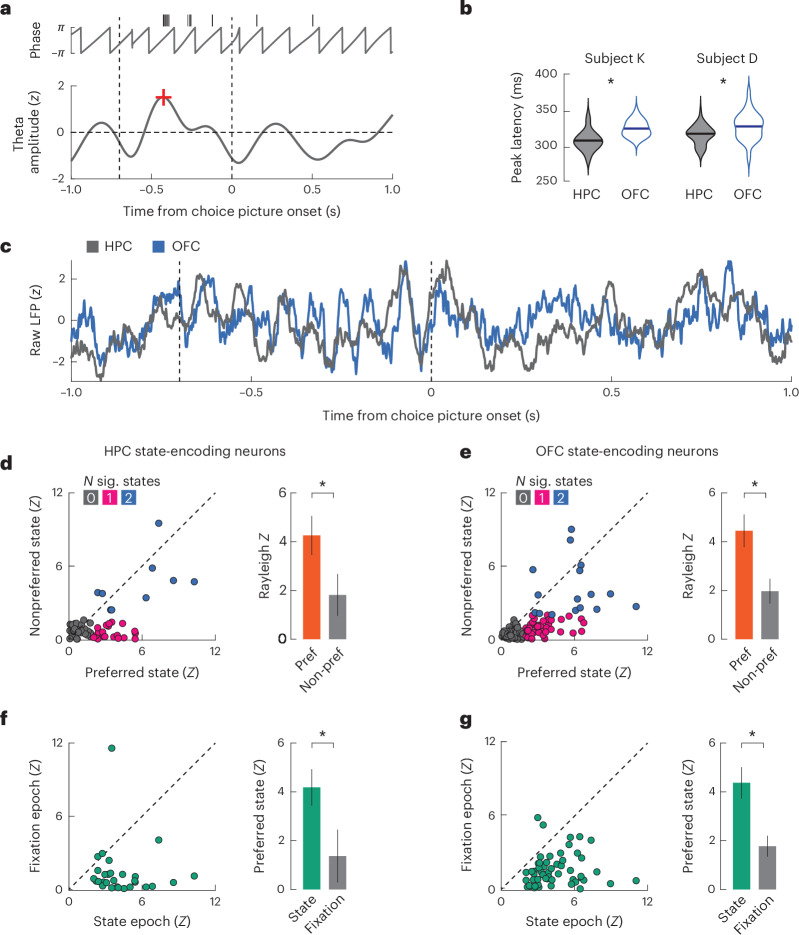
Phase modulation of single neurons. **a**, Example of phase locking from a single neuron on a single trial. Black tick marks indicate spikes. The red plus sign (+) denotes the first theta peak. Dashed vertical lines indicate state and choice epochs, respectively. This example is from the HPC in subject D. **b**, Mean time of the first peak of the theta rhythm following the onset of the state cue. The first theta peak occurred significantly earlier in the HPC than in the OFC in both subjects; *P < 0.001, two-sample two-sided *t*-test (subject K: *t*_528_ = 16.18, *P* = 4.0 × 10^−48^; subject D: *t*_404_ = 4.64, *P* = 4.7 × 10^−6^). **c**, Example raw LFP waveforms simultaneously recorded from the HPC and OFC during a single trial by subject K. Dashed vertical lines indicate state and choice epochs, respectively. **d**, Scatter plot comparing the degree of theta phase locking (Rayleigh *Z* statistic) during the state epoch for those 29 HPC neurons that significantly encoded state information. Neurons were significantly more phase locked during their preferred (pref) state. Each marker in the scatter plot denotes a single neuron. In the bar plots, the bars represent the mean and the error bars denote the bootstrapped 95% confidence intervals; *P < 0.001, paired *t*-test (*t*_28_ = 6.90, *P* = 1.7 × 10^−7^). **e**, Phase locking during the state epoch for 56 OFC neurons that encoded state information during the choice epoch. Neurons were significantly more phase locked during their preferred state. Each marker in the scatter plot denotes a single neuron. In the bar plots, the bars represent the mean, and the error bars denote the bootstrapped 95% confidence intervals; *P < 0.001, paired *t*-test (*t*_55_ = 8.30, *P* = 2.8 × 10^−11^). **f**, HPC state-encoding neurons exhibit greater phase modulation during the state epoch than during fixation epochs during trials of their preferred state. Each marker in the scatter plot denotes a single neuron. In the bar plots, the bars represent the mean, and the error bars denote the bootstrapped 95% confidence intervals; *P < 0.001, paired *t*-test (*t*_28_ = 4.96, *P* = 3.1 × 10^−5^). **g**, The 55 OFC neurons that encode state information during the choice epoch, but not the state epoch, show increased phase modulation in the state epoch relative to the fixation epoch during trials of their preferred state. Each marker in the scatter plot denotes a single neuron. In the bar plots, the bars represent the mean, and the error bars denote the bootstrapped 95% confidence intervals; *P < 0.001, paired *t*-test (*t*_55_ = 6.84, *P* = 6.8 × 10^−9^). Source data

Having established the presence of theta oscillations within our task, we next measured HPC–OFC communication via analysis of oscillatory coherence^25^. Theta coherence increased shortly after the onset of the state cue and peaked after the onset of choice options (Fig. 5c,e). A single-trial example of this synchronization is shown in Fig. 6c. Theta coherence did not differ between free and forced choice trials during these epochs (Fig. 6f). Not all theta events coincided with interareal synchronization. The onset of fixation elicited cross-trial theta phase alignment but no interareal theta coherence, whereas the onset of the state cue elicited both phenomena.

To quantify the direction of information flow between the OFC and HPC, we used generalized partial directed coherence (GPDC; see Methods). Across both subjects, we measured stronger HPC → OFC directionality than OFC → HPC directionality, most prominently in the theta band with a peak at 5 Hz (Supplementary Fig. 7). We confirmed this finding with a Granger causality analysis (Supplementary Fig. 7). In addition, the first theta peak during the state epoch occurred significantly earlier in the HPC than in the OFC in both animals (Fig. 6b), supporting the GPDC and Granger causality analyses.

We next examined whether HPC and OFC neurons were significantly modulated by local field potential (LFP) oscillatory activity during the state cue period (Fig. 6a). Many neurons in both brain areas and subjects were significantly theta modulated (subject K: OFC = 81/351 or 23%, HPC = 40/179 or 22%; subject D: OFC = 136/231 or 59%, HPC: 64/125 or 51%), whereas the proportion of neurons modulated by other frequency bands did not exceed chance levels (binomial test, *P* > 0.1 in all cases). Thus, the ongoing theta rhythm appears to organize the firing of HPC and OFC units during the period when theta coherence occurs between the regions. We then determined whether this phase modulation could be a candidate mechanism enabling the transfer of information from the HPC to the OFC.

We first quantified whether state-encoding hippocampal neurons were more likely to be phase modulated during trials of their preferred state than during trials in the other state (Methods). Owing to the relatively low numbers of state-encoding neurons in each animal, we pooled across animals for these analyses. State-encoding HPC neurons were significantly more phase modulated for their preferred state than for their nonpreferred state (Fig. 6d). They were also significantly more likely to be modulated in only one and not both states (expected proportion = 20/65, observed proportion = 9/65, *P* = 0.02, binomial test). We repeated these analyses for state-encoding neurons in the OFC (Fig. 6e). Because the OFC carried only a near-chance proportion of state-selective neurons during the state epoch, we focused on those OFC neurons that significantly encoded state during the choice epoch but not the state epoch. Half of these neurons (56/113 or 50%) were significantly phase modulated for at least one state during the state epoch. Moreover, these future state-encoding OFC neurons were significantly more strongly phase modulated during trials of their preferred state, and they were significantly less likely to show phase locking during both states than expected by chance (expected proportion = 27/113, observed proportion = 16/113, *P* = 0.02, binomial test). As an additional control, we compared phase modulation during the fixation epoch to phase modulation during the state epoch (Fig. 6f,g). Neurons that exhibited phase modulation during the state epoch were less likely to do so during the fixation epoch even though there was a significant response in the theta band during the fixation epoch (Fig. 5).

One issue with the interpretation of the results from the HPC is that they may have been confounded with firing rate. Neurons firing in their preferred state by definition fire more spikes, which gives more power to detect phase modulation. However, this explanation does not apply to the results from the OFC. The OFC neurons did not encode state information in their firing rates (Supplementary Fig. 8), but they did encode it in their phase. These findings suggest that phase coding, the organization of spikes with respect to coherent theta HPC–OFC oscillations, is a candidate mechanism enabling the transfer of state information from the HPC to the OFC to enable state-dependent valuation and choice.

## Discussion

Our results suggest that both the HPC and OFC are central to behaviors that depend on cognitive maps. HPC neurons group cues into behaviorally relevant categories that then select state-appropriate decision circuits in the OFC via theta synchronization. A major theory of OFC function is that it encodes an abstract ‘common currency’ value^24,26–28^. Our findings of distinct, state-specific subcircuits in the OFC challenge this notion. However, they also argue against the strict cognitive map hypothesis^9^, which argues that the critical function of the OFC is to encode the map itself. At the time of the state cue, when there is no value information present, the HPC appears to have the primary responsibility for encoding state information.

Instead, our results support a division of labor between the HPC and OFC^29^, whereby the HPC encodes task states and the relationships between them^20,30^ and the OFC uses those states to inform valuation and choice. An advantage of separating knowledge about the causal structure of the world from knowledge about value is that it allows rapid changes in action selection when values and goals change. For example, if we have knowledge of the various states involved in navigating the airport, when we experience a long line at security, we can flexibly update our goals, perhaps from getting a coffee after security to running to the gate to board our plane. Damage to the OFC typically produces deficits on tasks that require these kinds of inferences versus those that can be learned by trial and error^31,32^.

Although previous studies have varied the context of decisions, they did not observe a difference in the OFC value code^33,34^. For example, prior reports of ‘menu-invariant’ value coding in the OFC^33,34^ defined decision contexts according to differences in reward identities. For example, context A might involve choices between apple and cherry juice, whereas context B might involve choices between cherry and grape juice. A critical difference between this prior work and our own study is whether solving the task required the invoking of distinct task states in which the stimulus-value mappings were in conflict. Recent theoretical work argues that animals use Bayesian inference to determine whether some evidence is consistent with an existing state of the world or suggests the existence of a new state^20^. Their aim is to create a state transition graph that describes a behavioral task with a minimum number of necessary states^35,36^. A critical feature of our task is the conflict in choice–outcome contingencies across contexts necessitating the requirement of distinct states to solve the task.

Our task design also has similarities to occasion setting tasks, where cues signal whether responding to an upcoming stimulus will yield reinforcement^37^. However, there are some important differences. Occasion setting has primarily been associated with Pavlovian conditioning that does not readily translate to choice tasks, particularly a task such as ours where the reward contingencies are changing trial by trial. Indeed, Pavlovian occasion setting appears to depend on connections between the amygdala and the OFC rather than the HPC^38^. Despite similarities in structure to our task, occasion setting may involve a different type of learning that does not require a cognitive map.

Our findings are consistent with the increasing evidence that the HPC has a prominent role in reward-guided behavior. In rodents, investigators have decoded information from place cells to show that HPC neuronal activity tends to be biased toward goal locations^39,40^. In monkeys, HPC neurons have been found to encode specific value relationships between choice options^3^. Causal manipulations of the HPC also impair value-based decision-making, as shown by damage to the HPC in humans^41^ or electrical microstimulation of the HPC in monkeys^23^. In contrast to the HPC, the OFC has a long history of being associated with reward processing^13,42–44^. However, these studies have frequently emphasized that the OFC abstracts values across choice options to derive a common neural currency for value^13,15,28,45^. By contrast, our results suggest that the OFC value code is more flexible than previously described and can be reconfigured on the fly. Calculating these contextually appropriate values requires the coordinated interaction between HPC and OFC circuits, and our results support the notion that such communication occurs via oscillatory coherence in the theta band^23,25,46,47^.

Oscillatory coherence between structures has been proposed as a mechanism of information propagation through cell assembly formation^25^. For example, groups of OFC and HPC neurons that consistently fire near the trough of the theta oscillation will have mutually increased firing probabilities during periodic windows, increasing the opportunity for spike timing-dependent plasticity^48^. The cumulative effect will be increased covarying activity among these neurons, binding them into a functional assembly. In our task, the state cue elicited strong HPC-to-OFC theta coherence, and the timing of spikes of OFC state-encoding neurons was organized to this underlying rhythm during trials of their preferred state. Critically, the number of spikes emitted by these neurons did not change, consistent with a phase code in the absence of a rate code. Phase modulation could be a mechanism by which state-encoding HPC neurons can select specific cell assemblies in the OFC that will encode the relevant state-dependent value. We note that there are differences in the characteristics of theta between rodents and primates. In rodents, theta consists of a more prominent, frequency-restricted and continuous oscillation^49,50^. The theta signal in monkeys tends to be evoked by salient task events^51–54^ and then persists for several cycles of oscillation. For this reason, it is difficult to separate it cleanly into evoked and continuous components. Similar dynamics are observed in human recordings^53,55,56^. An interesting aspect of our results is that the theta response is evoked by the state cue but not by the choice options, which argues against a pure saliency explanation of the signal.

An outstanding question concerns the mechanisms that facilitate interareal synchronization. We found a dissociation between cross-trial theta phase alignment and interareal theta coherence. The onset of fixation elicited the former but not the latter, whereas the onset of the state cue elicited both phenomena. Neuromodulators may control whether the HPC and OFC synchronize. Local administration of dopamine in the prefrontal cortex rapidly increases theta coherence with the HPC^57^, whereas HPC projections to the ventral tegmental area have been theorized to allow important environmental information to elicit learning and memory processes^58^. This could help strengthen a subset of HPC–OFC synapses, enabling the generation of the state-dependent value code.

Another possibility is that a cortical area coordinates the interactions between the OFC and HPC. The HPC has generally been associated with learning new information and then passing this to the cortex^59,60^. Given that our animals are well trained on this task, we might anticipate another cortical area to be responsible for specifying the state information. Two regions of the prefrontal cortex may be particularly relevant. The lateral prefrontal cortex has a long history of involvement in specifying appropriate rules and contexts to guide behavior^61,62^, whereas more recent results from the medial prefrontal cortex have suggested that it may have an important role in specifying state transition graphs^7,8,63^.

A range of neuropsychiatric disorders are characterized by dysfunctional connectivity between the HPC and OFC^64,65^. A better understanding of what information is communicated between these structures, the context in which it occurs and the fundamental mechanisms enabling this communication could improve treatments that attempt to normalize activity within the circuit^66^.

## Methods

### Experimental model and subject details

All procedures were performed as specified in the National Research Council guidelines and were approved by the Animal Care and Use Committee at the University of California, Berkeley. Two male rhesus macaques (subjects D and K, respectively) aged 8 and 4 years and weighing 11 and 9 kg at the time of recording were used in the current study. Subjects sat in a primate chair (Crist Instruments), and eye movements were tracked with an infrared system (SR Research). Stimulus presentation and behavioral conditions were controlled using the NIMH MonkeyLogic toolbox^67^. Subjects had unilateral recording chambers implanted that allowed access to both the HPC and OFC.

### Task design

Stimuli were presented on a computer monitor positioned at a viewing distance of 30 cm. The subjects were trained to perform a state-dependent decision task. This required them to choose between eight reward-predictive pictures, where the reward amounts associated with the pictures depended on a cued task state (Fig. 1a). Subjects initiated trials by fixating on a white dot for 600 ms. After initial fixation, the subjects were shown a state cue that indicated whether the upcoming choice should be evaluated according to the value scheme associated with state A or B. After 600 ms, the state cue disappeared, and the subjects had to maintain central fixation for a further 100 ms. Finally, the choice options were presented. On 80% of the trials, two options were shown (free choice trials), whereas on the remaining 20% of the trials there was only one option (forced choice trials). The inclusion of forced choice trials ensured that subjects experienced all reward contingencies. Subjects reported their response by shifting their gaze to their selection and fixating for 300 ms. The values of the choice options corresponded to different amounts of apple juice reward.

To illustrate how state modulates value, and thus choice, consider a trial where the state cue was an orange fractal (indicating state A), and the choice options were the lollipop and postbox emojis. In state A, selecting the lollipop yielded four drops of juice, whereas selecting the postbox yielded only one drop of juice. However, had a state B cue been shown earlier (for example, a purple fractal), selecting the postbox would have yielded four drops of juice and the lollipop only one drop. State cues were pseudorandomized across trials, meaning that there was no serial dependence between trials. This design precluded the use of trial-and-error learning, and the optimal decision entirely depended on a given trial’s cued state. Subjects never choose between options with the same value; all other combinations of stimuli were used.

To unconfound neuronal activity related to the physical properties of the stimuli from the meaning they signified, we used two distinct cues for each state and value. Thus, we used two cues to indicate each state (four total state cues) and two pictures to indicate each possible value level (eight total choice options).

### Behavioral analysis

Behavioral accuracy was defined as choosing the more valuable option. To assess how decision times (DT) varied as a function of task parameters, we used the following regression model:

1 $$\begin{array}{rcl}{\rm{DT}}&=&{\beta }_{0}+{\beta }_{1}\times {\rm{state}}+{\beta }_{2}\times {\rm{abs}}\left(\Delta {\rm{value}}\right)\\ && +\,{\beta }_{3}\times {\rm{state}}\times \rm{{abs}}(\Delta {\rm{value}})+{\beta }_{4}\times {\rm{best}}\; {\rm{side}},\end{array}$$

where abs(Δvalue) indicates the absolute difference between the left and right choice options, state indicates the current task state (A or B), and best side indicates whether the best option was on either the right or left.

### Neurophysiological recordings

Subjects were fitted with titanium head positions and imaged in a 3T scanner. The resulting images were used to generate three-dimensional reconstructions of each subject’s skull and brain areas of interest. We then implanted custom radiotranslucent recording chambers made of polyether ether ketone (Gray Matter Research). Chambers were placed to allow neurophysiological recordings from the OFC and HPC.

In each recording session, we acutely lowered up to six Plexon V-probes (Plexon) distributed between the OFC and HPC. Each probe had either 32 or 64 contacts per probe with either 50- or 100-μm intercontact spacing. We used OnShape (PTC), a browser-based computer-assisted drawing software, to define electrode trajectories and design custom microdrives to acutely lower the electrodes. The custom drive assemblies were three-dimensionally printed using Rigid4000 Resin and a Form3+ printer (FormLabs).

Neuronal signals were acquired and digitized using a Plexon OmniPlex acquisition system with a digital head stage processor. Neuronal signals were acquired at 40 kHz, and LFPs were acquired at 1,000 Hz. Neurons were identified via manual spike sorting (Offline Sorter, Plexon) and retained if their mean firing rate over the recording session was at least 1 Hz.

### Single-neuron tuning

We used all free and forced choice trials for analyses unless specified otherwise. For analyses based on fixed time windows, we defined the fixation epoch as the 600 ms from the onset of fixation, the state epoch as the 400 ms that began 100 ms from the onset of the state cue and the choice epoch as the 400 ms that began 100 ms after the onset of the choice options.

To determine how state information evolved over the course of the trial, we performed a sliding-window two-way ANOVA for each neuron. Firing rates were smoothed using a bin width of 100 ms stepped by 25 ms. The factors of the ANOVA were state (A or B, coded as –1 and 1, respectively) and cue (which of the two state cues was presented, coded as –1 and 1, respectively). For each window, the proportion of neurons significant for the state term was assessed at *P* < 0.05. We compared across brain areas via *χ*^2^ tests assessed at *P* < 0.05.

To quantify the strength of the state tuning, we calculated *ω*_*p*_^2^ to estimate the effect size of the state factor in our sliding-window ANOVA models. *ω*_*p*_^2^ is a robust measure of effect size that adjusts for sample size and provides a less biased estimate of the proportion of variance attributable to a given factor^68,69^. We calculated *ω*_*p*_^2^ as

2 $${\omega }_{p}^{2}=\frac{{{\rm{DF}}}_{{{\rm{effect}}}}\times \left({{\rm{MS}}}_{{{\rm{effect}}}}-{{\rm{MS}}}_{{{\rm{error}}}}\right)}{{{\rm{DF}}}_{{{\rm{effect}}}}\times {{\rm{MS}}}_{{{\rm{effect}}}}+\left(N-{{\rm{DF}}}_{{{\rm{effect}}}}\right)\times {{\rm{MS}}}_{{{\rm{error}}}}},$$

where DF_effect_ indicates the degrees of freedom of a given ANOVA factor, MS_effect_ indicates the mean squared error associated with that factor, *N* indicates the total number of observations, and MS_error_ indicates the residual mean squared error.

To assess the unique contributions of value and state information during the choice epoch, we calculated the mean firing rate, *F*, and calculated the following linear regression:

3 $$F={\beta }_{0}+{\beta }_{1}\times {\rm{state}}+{\beta }_{2}\times {\rm{value}}+{\beta }_{3}\times {\rm{state}}\times {\rm{value}}+{\beta }_{4}\times t,$$

where state was a dummy coded variable that indicates which state was cued (A = –1 or B = 1), value indicates the amount of juice yielded by selecting a given option (coded as –2, –1, 1 and 2), and *t* is a nuisance parameter that indicates the trial number and was used to account for nonstationarity in neuronal recordings. Significance was assessed at *P* < 0.05.

To identify how neurons encoded value information, for each neuron, we performed the following linear regression on its activity during the choice epoch:

4 $$F={\beta }_{0}+{\beta }_{1}\times {\rm{value}}+{\beta }_{2}\times t,$$

where value indicates the number of drops of juice associated with the chosen option, and *t* is a nuisance parameter that indicates the trial number to account for nonstationarity. We performed this analysis separately for trials in which state A was in effect versus state B.

### Population state space analysis

We used principal component analysis (PCA) to study how populations of neurons represented values in each task state during the choice epoch. We constructed a state-value PC space based on the mean activity of each neuron in each task condition comprising the four values and two task states. We found that the first five PCs explained over 95% of the variance in both the OFC and HPC. In each of 1,000 iterations, we randomly selected 20 trials of each condition, obtained the mean firing rate of each neuron in each condition and projected those means into the previously established PC space. Within each bootstrap, our key analysis involved aligning value manifolds across task states via rigid rotation. The first step of the rotation procedure was to represent the values in each state as a vector and then calculate the angle between the two resulting vectors. After calculating the angle between the vectors representing the values in different task states, we used rotation to minimize the angle between the vectors, facilitating their alignment within PC space. To achieve this alignment, a rotation matrix was constructed based on the calculated angle between the vectors. This rotation matrix was applied to the vector representing task state B to align it with the vector representing task state A. The rotation was performed while preserving the shape of the vector, ensuring that the representation of neural activity corresponding to that task state remained consistent throughout the rotation process. By iteratively adjusting the rotation angle and applying the rotation matrix, the vectors representing different task states were progressively aligned in PC space. We quantified alignment via minimization of the root mean square error between points in the two value vectors.

### Comparing cross-state value generalization within and between brain areas

We used linear support vector machine (SVM) decoders (fitcecoc in MATLAB) to assess how value representations differed within and between task states and whether cross-state value decoding differed across brain areas. Given the near-optimal performance of the animals, we had few observations of the lowest value options being chosen. To overcome this class imbalance and avoid bias in the decoders, we used a bootstrapping procedure. Within each of 1,000 bootstraps, 20 trials of each value within each state were randomly selected (20 trials × 4 values × 2 states = 160 trials per bootstrap). Owing to the high feature-to-observation ratio, we used PCA to reduce dimensionality and only retained components that cumulatively explained 95% of the variance. PCA was performed across all 160 trials in each bootstrap to ensure a consistent PC space for subsequent decoding analyses.

We then trained and tested two SVM decoders, one for each state, using a stratified leave-one-out procedure where one trial of each value level was held out for testing. For instance, the state A SVM was trained on 76 trials (19 per value level), and within-state prediction accuracy was assessed on the held out 4 trials, 1 of each value level. A corresponding procedure was performed for the SVM trained on 76 trials (19 of each value level) in state B. Between-state prediction accuracy was assessed by testing the state A SVM on the 4 held out trials from the state B SVM and vice versa. This approach ensured that we always trained and tested on balanced sets of values within and between task states.

Because different numbers of neurons were recorded in the HPC and OFC, we could not directly compare their decoding accuracies when including all neurons. Therefore, we used a neuron-dropping approach where we repeated the analysis above with different numbers of neurons. We progressively added five neurons to each run of 1,000 bootstraps. Which subset of neurons was included in each run was also bootstrapped 1,000 times. This allowed us to compare across brain areas how the number of neurons affected within and between state value decoding.

### Cross-trial phase alignment

To measure cross-trial phase alignment, we extracted phase information from bandpass-filtered LFP signals using the Hilbert transform. Phase alignment strength was determined by calculating the mean resultant vector length, *R*, across trials using the following formula:

5 $$R=\frac{1}{\#{\mathrm{trials}}}\left|\mathop{\sum }\limits_{t=1}^{\#{\mathrm{trials}}}\exp (i\varphi ({\rm{f}},{\rm{t}}))\right|.$$

For each recording channel, *i*, this measures the degree to which a given phase, *φ*, of LFP frequency, *f*, is aligned across trials for each time point, *t*, in the trial. This analysis was performed separately for each recording site that had at least one well-isolated neuron (K: HPC = 179 channels and OFC = 351 channels; D: HPC = 125 channels and OFC = 281 channels).

### Interareal coherence

Time-resolved LFP power and interareal coherence were measured via multitaper coherograms^70^, which used three tapers, a 1-s time window and 90% overlap between windows. In multitaper spectral analysis, the ‘tapers’ are a set of orthogonal or nearly orthogonal functions (also known as ‘Slepian functions’) that are applied to the time series data before estimating the power spectral density. The Hamming window that is often used in the fast Fourier transform is an example of a taper. These tapers are designed to reduce spectral leakage and enhance the precision of time-resolved spectral estimates. By applying multiple tapers with slightly different spectral characteristics, the multitaper approach to signal processing allows for a more accurate estimation of the power spectral density and, in the case of coherograms, coherence between two signals.

We observed a high degree of redundancy of the LFP signal across the contacts of a single V probe. To prevent oversampling of the same underlying signal, we used a single channel per V probe. For each probe, we identified the channel that had the highest cross-trial phase alignment and at least one neuron. We then computed HPC–OFC coherences between this limited subset of channels. This restricted our analysis to 42 HPC–OFC channel pairs for subject D and 28 HPC–OFC channel pairs for subject K. To facilitate comparison across sessions, coherences were *z* scored for each HPC–OFC electrode pair.

Note that cross-trial phase alignment and interareal coherence are calculated with very different temporal resolutions, which allows for the kind of dissociation in these measures that we observed during the fixation epoch. Cross-trial phase alignment uses the Hilbert transform and is calculated each millisecond. By contrast, spectral coherence, being fast Fourier transform based, evaluates coherence over a much larger window. We used a 1-s window because this would enable us to capture at least three cycles of slow oscillations like theta and therefore only reflect truly ongoing synchronization between the HPC and OFC.

### GPDC

To assess the direction of HPC–OFC communication, we calculated the GPDC, a frequency-resolved estimate of Granger causality that uses multivariate autoregressive modeling to exploit the predictability of information in one brain area by past activity in another^71^. We fit GPDC models to the 600 ms that began 300 ms after the state cue onset, which corresponded to the period of rising theta coherence. Models were fit in both directions (HPC → OFC, OFC → HPC), and the difference was taken to obtain the net signal directionality. Significance was assessed by measuring whether the bootstrapped confidence intervals of the net signal directionality overlapped with zero.

### Phase modulation of single neurons

Phase modulation of HPC and OFC single neurons was assessed by examining the distribution of a given neuron’s spiking with respect to the phase angle of the simultaneously recorded LFP. Instantaneous LFP phase angles were determined by bandpass filtering the LFPs to a frequency of interest (theta (4–8 Hz), alpha (9–12 Hz), beta (13–30 Hz) and gamma (30–60 Hz)) and then extracting phase information via the Hilbert transform. We used Rayleigh’s test of circular uniformity to assess the extent to which spikes clustered at a specific phase angle compared to the same number of spikes being uniformly distributed across all phase angles. We assessed the phase modulation of each neuron during both the fixation and state epochs. Significant phase modulation was defined as *P* < 0.01 via a Rayleigh’s test.

We compared the degree of theta phase modulation for each neuron’s preferred and nonpreferred states. We defined each neurons’ preferred state by regressing *z* scored firing rates against the predictors of state (dummy coded as –1 for state A and 1 for state B) and cue type (dummy coded as –1 for set 1 and 2 for set 2). We determined the preferred state via the sign of the beta weight for the state factor. To determine whether neurons were more likely to exhibit phase modulation in only one and not both states, we calculated the expected proportion of neurons phase modulated in both states as the union of the probabilities of being phase modulated in either state. We then used a binomial test to determine whether the observed proportion was significantly different from the expected proportion.

### Identifying theta peaks within single trials

Theta peaks during the state epoch were identified by first bandpassing the HPC and OFC LFP channels in the theta band (4–8 Hz). Next, we extracted the amplitude of the bandpassed LFP via the Hilbert transform. We then *z* scored theta amplitudes on each channel and aligned them to the onset of the state cue within each individual trial. We defined each trial’s theta peak as the first positive peak of the single-trial theta amplitudes after state cue onset that was greater than the mean (*z* > 0). Only recording sites that had at least one well-isolated neuron were included in the analysis.

### Statistics

All statistical tests are described in the main text or the corresponding figure legends. Error bars and shading indicate 95% confidence intervals unless otherwise specified. We used a bootstrapping procedure to estimate confidence intervals. This procedure involved randomly subsampling 80% of the data and then computing the relevant statistic. This process was repeated 1,000 times, each time randomly subsampling the data. The 5th and 95th percentiles of the resulting distributions were then used as confidence intervals. All terms in regression and ANOVA models were normalized and had maximum variance inflation factors of 1.7. All comparisons were two sided.

### Reporting summary

Further information on research design is available in the Nature Portfolio Reporting Summary linked to this article.

## Online content

Any methods, additional references, Nature Portfolio reporting summaries, source data, extended data, supplementary information, acknowledgements, peer review information; details of author contributions and competing interests; and statements of data and code availability are available at 10.1038/s41593-024-01839-5.

## Supplementary information

Supplementary Information Supplementary Figs. 1–8 along with corresponding legends and captions.

Reporting Summary

## Source data

Source Data Statistical source data for Figs. 1–6.

## Data Availability

The dataset supporting the current work is available from the corresponding author on request. Source data are provided with this paper.

## Code availability

The code developed to analyze the data is available at https://github.com/t-elston/cued_flex_val.

## References

[1] O’Keefe, J. & Nadel, L. *The Hippocampus as a Cognitive Map* (Oxford Univ. Press, 1978).

[2] Behrens, T. E. J. et al. What is a cognitive map? Organizing knowledge for flexible behavior. *Neuron* **100**, 490–509 (2018). 30359611 10.1016/j.neuron.2018.10.002

[3] Knudsen, E. B. & Wallis, J. D. Hippocampal neurons construct a map of an abstract value space. *Cell* **184**, 4640–4650 (2021). 34348112 10.1016/j.cell.2021.07.010 PMC8459666

[4] Whittington, J. C. R. et al. The Tolman–Eichenbaum machine: unifying space and relational memory through generalization in the hippocampal formation. *Cell* **183**, 1249–1263 (2020). 33181068 10.1016/j.cell.2020.10.024 PMC7707106

[5] Whittington, J. C. R., McCaffary, D., Bakermans, J. J. W. & Behrens, T. E. J. How to build a cognitive map. *Nat. Neurosci.* **25**, 1257–1272 (2022). 36163284 10.1038/s41593-022-01153-y

[6] Schuck, N. W., Cai, M. B., Wilson, R. C. & Niv, Y. Human orbitofrontal cortex represents a cognitive map of state space. *Neuron* **91**, 1402–1412 (2016). 27657452 10.1016/j.neuron.2016.08.019 PMC5044873

[7] Baram, A. B., Muller, T. H., Nili, H., Garvert, M. M. & Behrens, T. E. J. Entorhinal and ventromedial prefrontal cortices abstract and generalize the structure of reinforcement learning problems. *Neuron* **109**, 713–723 (2021). 33357385 10.1016/j.neuron.2020.11.024 PMC7889496

[8] Park, S. A., Miller, D. S., Nili, H., Ranganath, C. & Boorman, E. D. Map making: constructing, combining, and inferring on abstract cognitive maps. *Neuron* **107**, 1226–1238 (2020). 32702288 10.1016/j.neuron.2020.06.030 PMC7529977

[9] Niv, Y. Learning task-state representations. *Nat. Neurosci.* **22**, 1544–1553 (2019). 31551597 10.1038/s41593-019-0470-8 PMC7241310

[10] Basu, R. et al. The orbitofrontal cortex maps future navigational goals. *Nature* **599**, 449–452 (2021). 34707289 10.1038/s41586-021-04042-9 PMC8599015

[11] Zhou, J. et al. Complementary task structure representations in hippocampus and orbitofrontal cortex during an odor sequence task. *Curr. Biol.* **29**, 3402–3409 (2019). 31588004 10.1016/j.cub.2019.08.040 PMC6810873

[12] Zhou, J. et al. Evolving schema representations in orbitofrontal ensembles during learning. *Nature* **590**, 606–611 (2021). 33361819 10.1038/s41586-020-03061-2 PMC7906913

[13] Padoa-Schioppa, C. & Assad, J. A. Neurons in the orbitofrontal cortex encode economic value. *Nature* **441**, 223–226 (2006). 16633341 10.1038/nature04676 PMC2630027

[14] Cai, X. & Padoa-Schioppa, C. Contributions of orbitofrontal and lateral prefrontal cortices to economic choice and the good-to-action transformation. *Neuron* **81**, 1140–1151 (2014). 24529981 10.1016/j.neuron.2014.01.008 PMC3951647

[15] Kennerley, S. W., Dahmubed, A. F., Lara, A. H. & Wallis, J. D. Neurons in the frontal lobe encode the value of multiple decision variables. *J. Cogn. Neurosci.* **21**, 1162–1178 (2009). 18752411 10.1162/jocn.2009.21100 PMC2715848

[16] Wallis, J. D. & Rich, E. L. Challenges of interpreting frontal neurons during value-based decision-making. *Front. Neurosci.* **5**, 124 (2011). 22125508 10.3389/fnins.2011.00124 PMC3222102

[17] Balewski, Z. Z., Elston, T. W., Knudsen, E. B. & Wallis, J. D. Value dynamics affect choice preparation during decision-making. *Nat. Neurosci.* **26**, 1575–1583 (2023). 37563295 10.1038/s41593-023-01407-3 PMC10576429

[18] Eichenbaum, H. On the integration of space, time, and memory. *Neuron* **95**, 1007–1018 (2017). 28858612 10.1016/j.neuron.2017.06.036 PMC5662113

[19] McKenzie, S. et al. Hippocampal representation of related and opposing memories develop within distinct, hierarchically organized neural schemas. *Neuron* **83**, 202–215 (2014). 24910078 10.1016/j.neuron.2014.05.019 PMC4082468

[20] Sanders, H., Wilson, M. A. & Gershman, S. J. Hippocampal remapping as hidden state inference. *eLife* **9**, e51140 (2020). 32515352 10.7554/eLife.51140 PMC7282808

[21] Barbas, H. & Blatt, G. J. Topographically specific hippocampal projections target functionally distinct prefrontal areas in the rhesus monkey. *Hippocampus* **5**, 511–533 (1995). 8646279 10.1002/hipo.450050604

[22] Carmichael, S. T. & Price, J. L. Limbic connections of the orbital and medial prefrontal cortex in macaque monkeys. *J. Comp. Neurol.* **363**, 615–641 (1995). 8847421 10.1002/cne.903630408

[23] Knudsen, E. B. & Wallis, J. D. Closed-loop theta stimulation in the orbitofrontal cortex prevents reward-based learning. *Neuron* **106**, 537–547 (2020). 32160515 10.1016/j.neuron.2020.02.003 PMC7480400

[24] Padoa-Schioppa, C. Neurobiology of economic choice: a good-based model. *Annu. Rev. Neurosci.* **34**, 333–359 (2011). 21456961 10.1146/annurev-neuro-061010-113648 PMC3273993

[25] Fries, P. Rhythms for cognition: communication through coherence. *Neuron* **88**, 220–235 (2015). 26447583 10.1016/j.neuron.2015.09.034 PMC4605134

[26] Padoa-Schioppa, C. & Conen, K. E. Orbitofrontal cortex: a neural circuit for economic decisions. *Neuron* **96**, 736–754 (2017). 29144973 10.1016/j.neuron.2017.09.031 PMC5726577

[27] Wallis, J. D. Cross-species studies of orbitofrontal cortex and value-based decision-making. *Nat. Neurosci.* **15**, 13–19 (2012). 10.1038/nn.2956 PMC3549638 22101646

[28] Levy, D. J. & Glimcher, P. W. The root of all value: a neural common currency for choice. *Curr. Opin. Neurobiol.* **22**, 1027–1038 (2012). 22766486 10.1016/j.conb.2012.06.001 PMC4093837

[29] Knudsen, E. B. & Wallis, J. D. Taking stock of value in the orbitofrontal cortex. *Nat. Rev. Neurosci.* **23**, 428–438 (2022). 35468999 10.1038/s41583-022-00589-2 PMC10511019

[30] Baraduc, P., Duhamel, J. R. & Wirth, S. Schema cells in the macaque hippocampus. *Science* **363**, 635–639 (2019). 30733419 10.1126/science.aav5404

[31] Gremel, C. M. & Costa, R. M. Orbitofrontal and striatal circuits dynamically encode the shift between goal-directed and habitual actions. *Nat. Commun.* **4**, 2264 (2013). 23921250 10.1038/ncomms3264 PMC4026062

[32] McDannald, M. A., Lucantonio, F., Burke, K. A., Niv, Y. & Schoenbaum, G. Ventral striatum and orbitofrontal cortex are both required for model-based, but not model-free, reinforcement learning. *J. Neurosci.* **31**, 2700–2705 (2011). 21325538 10.1523/JNEUROSCI.5499-10.2011 PMC3079289

[33] Padoa-Schioppa, C. & Assad, J. A. The representation of economic value in the orbitofrontal cortex is invariant for changes of menu. *Nat. Neurosci.* **11**, 95–102 (2008). 18066060 10.1038/nn2020 PMC2646102

[34] Xie, J. & Padoa-Schioppa, C. Neuronal remapping and circuit persistence in economic decisions. *Nat. Neurosci.* **19**, 855–861 (2016). 27159800 10.1038/nn.4300 PMC4882218

[35] Courville, A. C., Daw, N. D. & Touretzky, D. S. Bayesian theories of conditioning in a changing world. *Trends Cogn. Sci.* **10**, 294–300 (2006). 16793323 10.1016/j.tics.2006.05.004

[36] Gershman, S. J., Blei, D. M. & Niv, Y. Context, learning, and extinction. *Psychol. Rev.* **117**, 197–209 (2010). 20063968 10.1037/a0017808

[37] Fraser, K. M. & Holland, P. C. Occasion setting. *Behav. Neurosci.* **133**, 145–175 (2019). 30907616 10.1037/bne0000306 PMC6447318

[38] Fraser, K. M. & Janak, P. H. Basolateral amygdala and orbitofrontal cortex, but not dorsal hippocampus, are necessary for the control of reward-seeking by occasion setters. *Psychopharmacology* **240**, 623–635 (2023). 36056949 10.1007/s00213-022-06227-0 PMC9931670

[39] Pfeiffer, B. E. & Foster, D. J. Hippocampal place-cell sequences depict future paths to remembered goals. *Nature* **497**, 74–79 (2013). 23594744 10.1038/nature12112 PMC3990408

[40] Wikenheiser, A. M. & Redish, A. D. Hippocampal theta sequences reflect current goals. *Nat. Neurosci.* **18**, 289–294 (2015). 25559082 10.1038/nn.3909 PMC4428659

[41] Bakkour, A. et al. The hippocampus supports deliberation during value-based decisions. *eLife* **8**, e46080 (2019). 31268419 10.7554/eLife.46080 PMC6693920

[42] Rosenkilde, C. E., Bauer, R. H. & Fuster, J. M. Single cell activity in ventral prefrontal cortex of behaving monkeys. *Brain Res.* **209**, 375–394 (1981). 7225799 10.1016/0006-8993(81)90160-8

[43] Schoenbaum, G., Chiba, A. A. & Gallagher, M. Orbitofrontal cortex and basolateral amygdala encode expected outcomes during learning. *Nat. Neurosci.* **1**, 155–159 (1998). 10195132 10.1038/407

[44] Wallis, J. D. & Miller, E. K. Neuronal activity in primate dorsolateral and orbital prefrontal cortex during performance of a reward preference task. *Eur. J. Neurosci.* **18**, 2069–2081 (2003). 14622240 10.1046/j.1460-9568.2003.02922.x

[45] Montague, P. R. & Berns, G. S. Neural economics and the biological substrates of valuation. *Neuron* **36**, 265–284 (2002). 12383781 10.1016/s0896-6273(02)00974-1

[46] Riceberg, J. S., Srinivasan, A., Guise, K. G. & Shapiro, M. L. Hippocampal signals modify orbitofrontal representations to learn new paths. *Curr. Biol.* **32**, 3407–3413 (2022). 35764092 10.1016/j.cub.2022.06.010 PMC11073633

[47] Jones, M. W. & Wilson, M. A. Theta rhythms coordinate hippocampal–prefrontal interactions in a spatial memory task. *PLoS Biol.* **3**, e402 (2005). 16279838 10.1371/journal.pbio.0030402 PMC1283536

[48] Caporale, N. & Dan, Y. Spike timing-dependent plasticity: a Hebbian learning rule. *Annu. Rev. Neurosci.* **31**, 25–46 (2008). 18275283 10.1146/annurev.neuro.31.060407.125639

[49] Buzsaki, G. Theta oscillations in the hippocampus. *Neuron* **33**, 325–340 (2002). 11832222 10.1016/s0896-6273(02)00586-x

[50] Vanderwolf, C. H. Hippocampal electrical activity and voluntary movement in the rat. *Electroencephalogr. Clin. Neurophysiol.* **26**, 407–418 (1969). 4183562 10.1016/0013-4694(69)90092-3

[51] Mao, D. et al. Spatial modulation of hippocampal activity in freely moving macaques. *Neuron* **109**, 3521–3534 (2021). 34644546 10.1016/j.neuron.2021.09.032 PMC8571030

[52] Stewart, M. & Fox, S. E. Hippocampal theta activity in monkeys. *Brain Res.* **538**, 59–63 (1991). 2018932 10.1016/0006-8993(91)90376-7

[53] Zhu, S. L., Lakshminarasimhan, K. J. & Angelaki, D. E. Computational cross-species views of the hippocampal formation. *Hippocampus* **33**, 586–599 (2023). 37038890 10.1002/hipo.23535 PMC10947336

[54] Courellis, H. S. et al. Spatial encoding in primate hippocampus during free navigation. *PLoS Biol.* **17**, e3000546 (2019). 31815940 10.1371/journal.pbio.3000546 PMC6922474

[55] Ekstrom, A. D. et al. Human hippocampal theta activity during virtual navigation. *Hippocampus* **15**, 881–889 (2005). 16114040 10.1002/hipo.20109

[56] Watrous, A. J. et al. A comparative study of human and rat hippocampal low-frequency oscillations during spatial navigation. *Hippocampus* **23**, 656–661 (2013). 23520039 10.1002/hipo.22124 PMC4068262

[57] Benchenane, K. et al. Coherent theta oscillations and reorganization of spike timing in the hippocampal–prefrontal network upon learning. *Neuron* **66**, 921–936 (2010). 20620877 10.1016/j.neuron.2010.05.013

[58] Lisman, J. E. & Grace, A. A. The hippocampal–VTA loop: controlling the entry of information into long-term memory. *Neuron* **46**, 703–713 (2005). 15924857 10.1016/j.neuron.2005.05.002

[59] Squire, L. R. Memory and the hippocampus: a synthesis from findings with rats, monkeys, and humans. *Psychol. Rev.* **99**, 195–231 (1992). 1594723 10.1037/0033-295x.99.2.195

[60] Squire, L. R. & Alvarez, P. Retrograde amnesia and memory consolidation: a neurobiological perspective. *Curr. Opin. Neurobiol.* **5**, 169–177 (1995). 7620304 10.1016/0959-4388(95)80023-9

[61] Wallis, J. D., Anderson, K. C. & Miller, E. K. Single neurons in prefrontal cortex encode abstract rules. *Nature* **411**, 953–956 (2001). 11418860 10.1038/35082081

[62] Miller, E. K. & Cohen, J. D. An integrative theory of prefrontal cortex function. *Annu. Rev. Neurosci.* **24**, 167–202 (2001). 11283309 10.1146/annurev.neuro.24.1.167

[63] Maggi, S. & Humphries, M. D. Activity subspaces in medial prefrontal cortex distinguish states of the world. *J. Neurosci.* **42**, 4131–4146 (2022). 35422440 10.1523/JNEUROSCI.1412-21.2022 PMC9121833

[64] Godsil, B. P., Kiss, J. P., Spedding, M. & Jay, T. M. The hippocampal–prefrontal pathway: the weak link in psychiatric disorders? *Eur. Neuropsychopharmacol.* **23**, 1165–1181 (2013). 23332457 10.1016/j.euroneuro.2012.10.018

[65] Small, S. A., Schobel, S. A., Buxton, R. B., Witter, M. P. & Barnes, C. A. A pathophysiological framework of hippocampal dysfunction in ageing and disease. *Nat. Rev. Neurosci.* **12**, 585–601 (2011). 21897434 10.1038/nrn3085 PMC3312472

[66] Creed, M., Pascoli, V. J. & Luscher, C. Addiction therapy. Refining deep brain stimulation to emulate optogenetic treatment of synaptic pathology. *Science* **347**, 659–664 (2015). 25657248 10.1126/science.1260776

[67] Hwang, J., Mitz, A. R. & Murray, E. A. NIMH MonkeyLogic: behavioral control and data acquisition in MATLAB. *J. Neurosci. Methods* **323**, 13–21 (2019). 31071345 10.1016/j.jneumeth.2019.05.002 PMC6743332

[68] Olejnik, S. & Algina, J. Generalized eta and omega squared statistics: measures of effect size for some common research designs. *Psychol. Methods* **8**, 434–447 (2003). 14664681 10.1037/1082-989X.8.4.434

[69] Lakens, D. Calculating and reporting effect sizes to facilitate cumulative science: a practical primer for *t*-tests and ANOVAs. *Front. Psychol.* **4**, 863 (2013). 24324449 10.3389/fpsyg.2013.00863 PMC3840331

[70] Bokil, H., Tchernichovsky, O. & Mitra, P. P. Dynamic phenotypes: time series analysis techniques for characterizing neuronal and behavioral dynamics. *Neuroinformatics* **4**, 119–128 (2006). 16595862 10.1385/NI:4:1:119

[71] Baccala, L. A., Takahashi, D. Y. & Sameshima, K. Generalized partial directed coherence. In *Proc. 15th International Conference on Digital Signal Processing* 162–166 (IEEE, 2007).

