# Supplementary material for: Context-dependent decision-making in the primate hippocampal–prefrontal circuit
Source: Nat Neurosci. 2025 Jan 6;28(2):374–82. doi: 10.1038/s41593-024-01839-5 (PMC11802454; doi:10.1038/s41593-024-01839-5)
Supplement: Supplementary file 1 — Supplementary Figs. 1–8 along with corresponding legends and captions. [file 41593_2024_1839_MOESM1_ESM.pdf]

nature neuroscience

Supplementary information https://doi.org/10.1038/s41593-024-01839-5

Context-dependent decision-making in the primate hippocampal–prefrontal circuit

In the format provided by the authors and unedited

Supplementary information

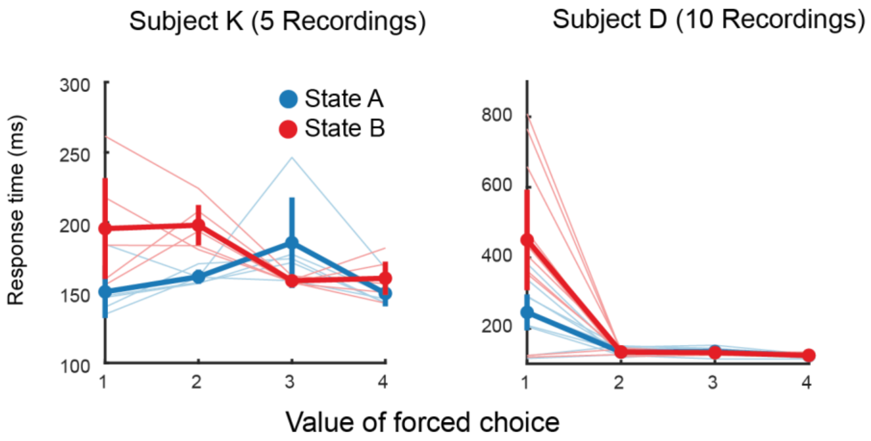

Figure S1. Choice response times during forced choice trials.

Thin lines are single sessions and thick lines are the mean. The error bars represent bootstrapped 95% confidence intervals. We fit a general linear regression model to each subject's data with predictors of Value and State. In both subjects, response times tended to decrease for higher value options (subject K: β = -6.3, p = 0.08; subject D: β = -67, p = 2.78 x 10^{-8}). Both subjects were also significantly faster at responding for state A choices (subject K: β = 29, p =0.005; subject D: β = 102, p = 0.0009). These effects also resulted in a significant State x Value interaction in both subjects (subject K: β = -8.3, p = 0.02; subject D: β = -31, p = 0.005). N = 5 recordings in subject K and N = 10 recordings for subject D. P-values were evaluated using two-tailed tests.

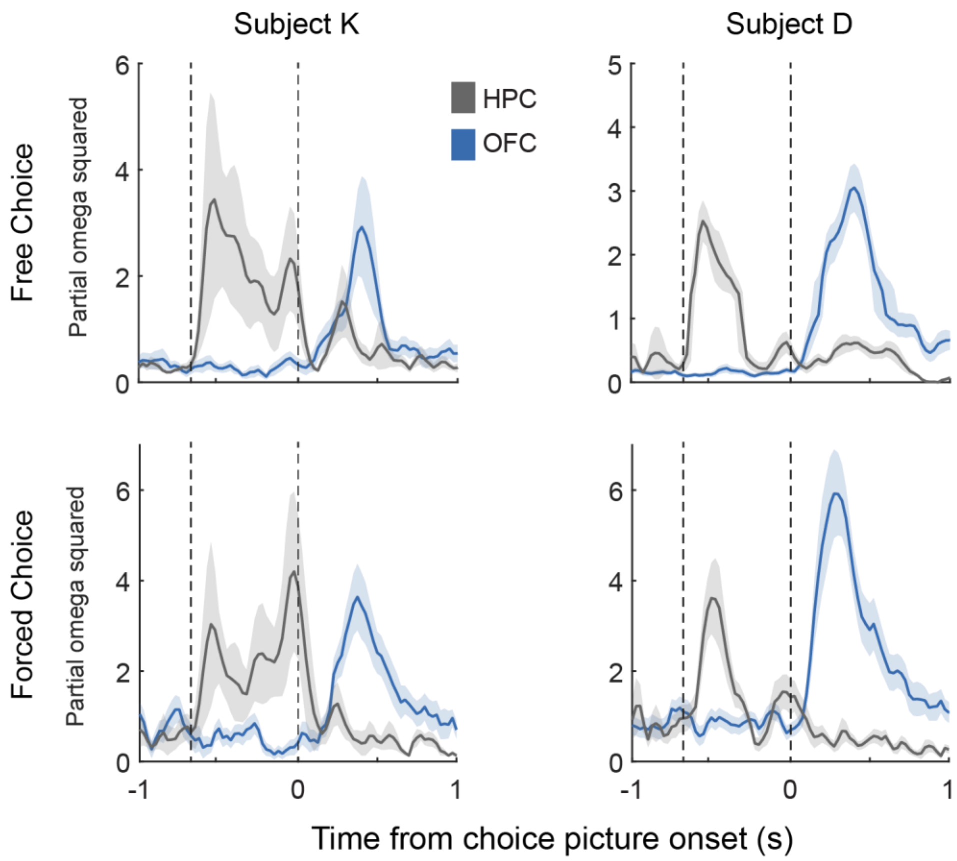

Figure S2. The strength of state-encoding in HPC and OFC during free and forced choice trials.

Neuronal selectivity was measured by partial omega squared, an unbiased measure of effect size. Values are expressed as percent explained variance. Dashed vertical lines indicate the state and choice epochs, respectively. The thick colored lines and shaded regions denote the mean and bootstrapped 95% confidence intervals.

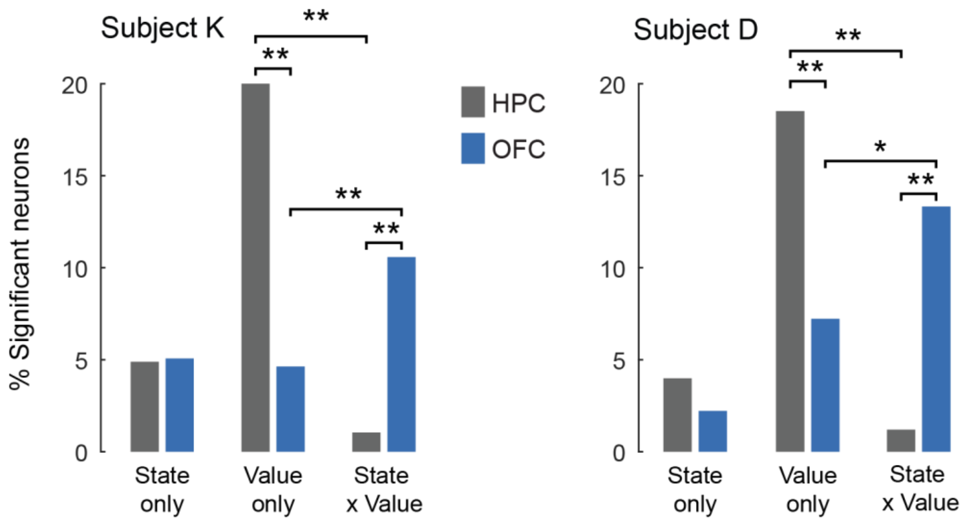

Figure S3. Single neuron tuning during the choice epoch.

In both subjects, HPC neurons tended to encode value independent of state, whereas OFC neurons encoded the interaction between the two parameters. * = $p < 0.05$, **$p < 0.005$, two-sided χ^2 tests. Subject K: HPC neurons were more likely than OFC neurons to encode only the value main effect (33/179 HPC vs 17/351 OFC, X2 = 25.63, p < .005). Conversely, OFC neurons were more likely than HPC neurons to encode the interaction between state and value (2/179 HPC vs 38/351, X2 = 16.01, p < .005). HPC neurons were more likely to encode only the value main effect than only the interaction effect (33/179 vs 2/179, X2 = 30.43, p < .005) whereas OFC neurons were more likely to encode only the interaction effect compared to only the value main effect (17/351 vs 38/351, = 8.70, p = .003).

Subject D: HPC neurons were more likely than OFC neurons to encode only the value main effect (30/125 HPC vs 29/281 OFC, X2 = 13.04, p < .005). Conversely, OFC neurons were more likely than HPC neurons to encode the interaction between state and value (0/125 HPC vs 46/281, X2 = 23.08, p < .005). HPC neurons were more likely to encode only the value main effect than only the interaction effect (30/125 vs 0/125, X2 = 34.09, p < .005) whereas OFC neurons were more likely to encode only the interaction effect compared to only the value main effect (29/281 vs 46/281, X2 = 4.45, p = .03).

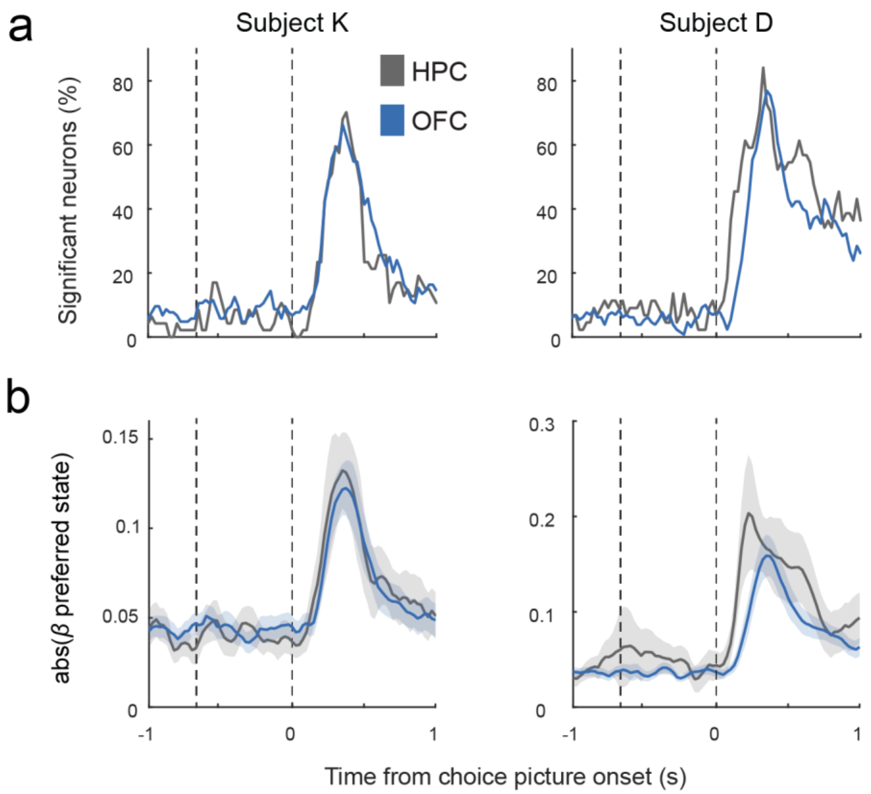

Figure S4. Time course of value coding in HPC and OFC.

(a) Time course of value encoding in HPC and OFC. At each time point, we regressed chosen value against binned firing rates (binwidth = 100 ms) for each state and plotted the proportion of neurons that significantly encoded value in at least one state. Dashed vertical lines indicate the onset of the state and choice epochs, respectively.

(b) Time course of value encoding in the neurons from (a). Plots indicate the absolute beta from neurons' preferred state, defined as the state that elicited the highest beta value. Thick lines denote means and shaded regions indicate bootstrapped 95% confidence intervals. Other conventions as in (a). To determine the latency of a signal, it is important to account for the strength of that signal, since a stronger signal can be detected more readily and therefore earlier. To account for this, we performed a one-way ANCOVA where we asked whether one brain area encoded value before the other while controlling for the strength of value tuning. There was no effect of brain area in subject K ($F_{1,149}$ = 0.61, p = 0.44), but in subject D, value was encoded significantly earlier in HPC than OFC ($F_{1,173}$ = 8.7, p < 0.003). This is consistent with HPC sending value-related information to OFC. Alternatively, it may reflect that a state-dependent value code (as in OFC) requires more computational processing than a general value code (as in HPC).

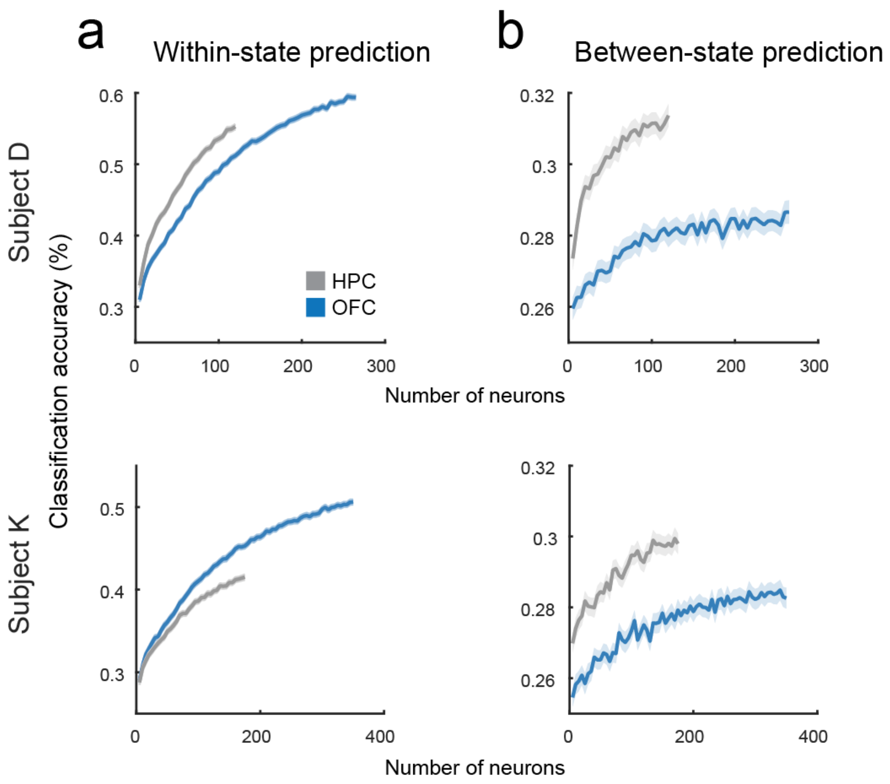

Figure S5. Decoding value within and between states in HPC and OFC as a function of neuron number.

a) Within-state value decoding. Classification accuracy as a function of the number of neurons when the decoder was tested within the same state it was trained (e.g. train on state A, test on state A). The thick lines denote the mean over 1000 bootstraps and the error bars denote the 95% confidence intervals derived from those 1000 bootstraps.

b) Between-state value decoding. Classification accuracy when the decoder was trained in one state and then tested in the other (e.g. train in state A and test in state B). Conventions as in (a).

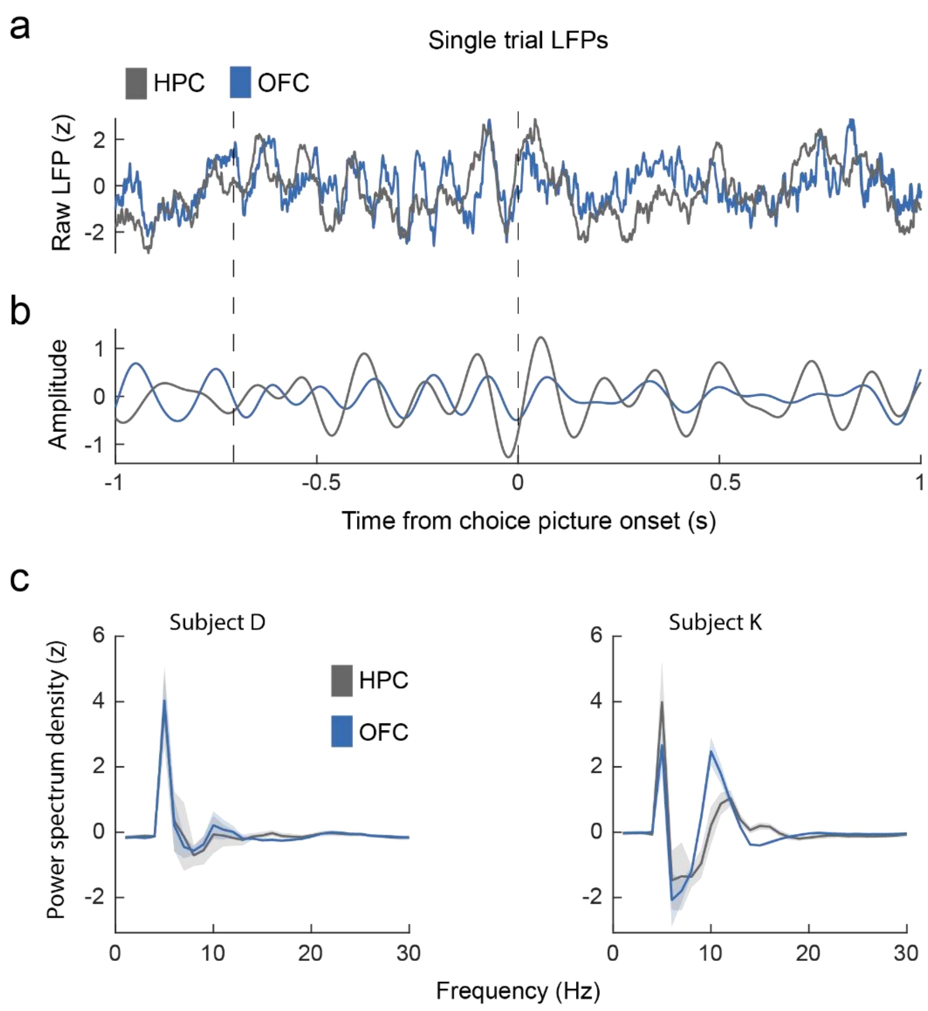

Figure S6. Example HPC and OFC LFP signals and power spectrum densities.

(a) An example LFP signal from HPC and OFC on one trial. Dashed vertical lines indicate the onset of the state and choice epochs, respectively.

(b) Same signal as in (a) band passed in the theta (4-8 Hz) range.

(c) Mean HPC and OFC power spectrum densities for each subject calculated using Welch's method for each trial separately and then averaged across trials for each channel. We used a 1 s reading window with 90% overlap between adjacent windows at a sampling rate of 1000 Hz. The aperiodic (1/f) component was estimated by fitting and subtracting a 3rd order polynomial. Thick lines denote the mean and shaded regions denote the 95% bootstrapped confidence intervals. There is significant theta power in both areas and both subjects. We also observed significant alpha power in one subject, but given its inconsistency between subjects, our analysis focused on the theta signal.

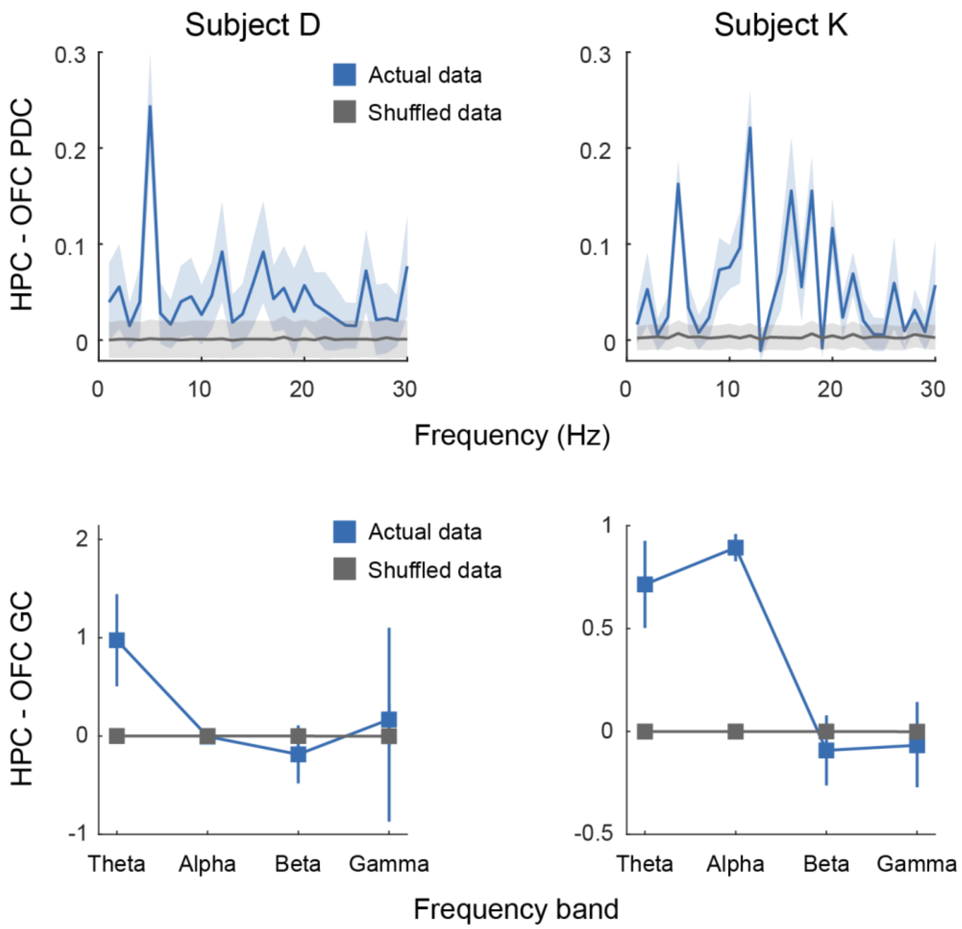

Figure S7. Directionality between HPC and OFC LFP signals

HPC-OFC LFP GPDC (top) and Granger causality (bottom) were evaluated over a 600 ms epoch that began 300 ms from the onset of the state cue. We selected this window as it corresponded to the time of elevated theta coherence (Fig. 6c). We computed models in both directions (HPC→OFC and OFC→HPC) and took the difference between them such that positive values indicate HPC→OFC directionality. GPDC and Granger causality were computed for each trial for each electrode pair and then averaged over the session. Across both subjects and measures, we observed stronger HPC→OFC directionality than OFC→HPC directionality. This was most prominent in the theta band for subject D, and the theta and alpha band for subject K. Although there was significant HPC→OFC directionality in the alpha band of subject K, there was no significant interareal coherence in alpha (Fig. 6c), so it is difficult to interpret this effect. The lines and error bars denote the mean and bootstrapped 95% confidence intervals. N = 42 channel pairs for subject D; N = 28 channel pairs for subject K.

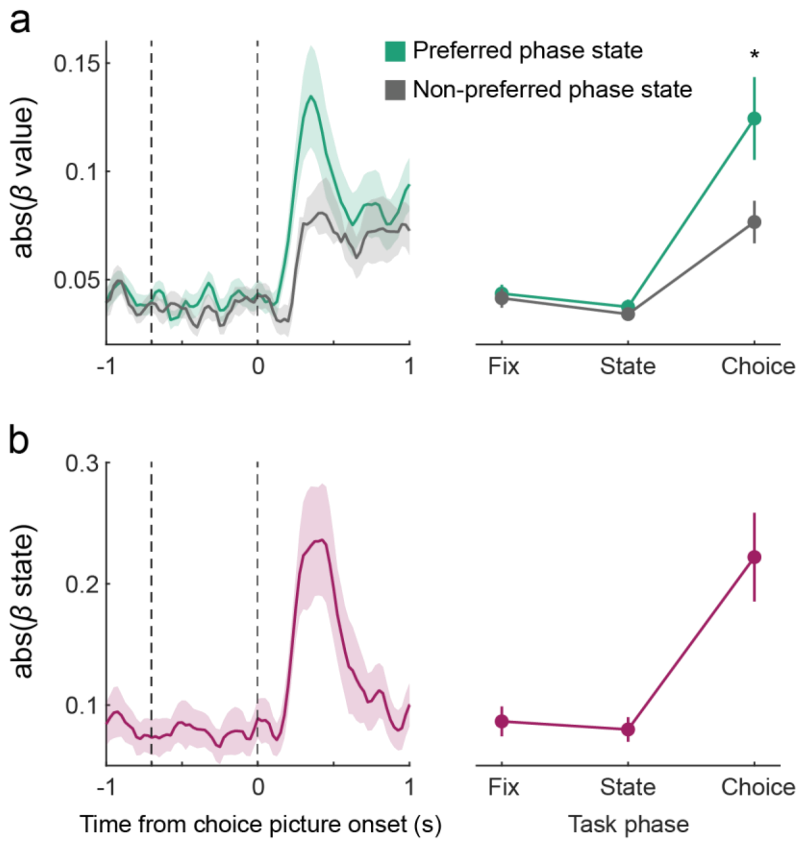

Figure S8. Time course of value and state tuning for the phase-modulated OFC state neurons.

(a) Left: Time course of the unsigned value-in-state beta weights for the 38 OFC neurons that encoded state information during the choice epoch and exhibited phase-modulation in only one state during the cue epoch. Dashed vertical lines indicate the onset of the state and choice epochs, respectively. Thick lines denote the mean and shaded regions denote the standard error of the mean. Right: Mean unsigned betas weights during each task epoch. Error bars denote standard error of the mean. * = p = 0.03, one-sided paired t-test ($t(26)$ = 1.89, p = .03).

(b) Time course of the unsigned state beta weights for the same neurons as in (a). Firing rates do not differentiate the state during the state epoch. Conventions as in (a).
