# Supplementary material for: Context-dependent decision-making in the primate hippocampal–prefrontal circuit
Source: Nat Neurosci. 2025 Jan 6;28(2):374–82. doi: 10.1038/s41593-024-01839-5 (PMC11802454; doi:10.1038/s41593-024-01839-5)
Supplement: Supplementary file 2 — Reporting Summary [file 41593_2024_1839_MOESM2_ESM.pdf]

nature portfolio

Corresponding author(s): Thomas Elston

Last updated by author(s): Sep 26, 2024

Reporting Summary

Nature Portfolio wishes to improve the reproducibility of the work that we publish. This form provides structure for consistency and transparency in reporting. For further information on Nature Portfolio policies, see our Editorial Policies and the Editorial Policy Checklist.

Statistics

For all statistical analyses, confirm that the following items are present in the figure legend, table legend, main text, or Methods section.

n/a	Confirmed	
☐	☒	The exact sample size (*n*) for each experimental group/condition, given as a discrete number and unit of measurement
☐	☒	A statement on whether measurements were taken from distinct samples or whether the same sample was measured repeatedly
☐	☒	The statistical test(s) used AND whether they are one- or two-sided<br>*Only common tests should be described solely by name; describe more complex techniques in the Methods section.*
☐	☒	A description of all covariates tested
☐	☒	A description of any assumptions or corrections, such as tests of normality and adjustment for multiple comparisons
☐	☒	A full description of the statistical parameters including central tendency (e.g. means) or other basic estimates (e.g. regression coefficient) AND variation (e.g. standard deviation) or associated estimates of uncertainty (e.g. confidence intervals)
☐	☒	For null hypothesis testing, the test statistic (e.g. *F*, *t*, *r*) with confidence intervals, effect sizes, degrees of freedom and *P* value noted<br>*Give P values as exact values whenever suitable.*
☒	☐	For Bayesian analysis, information on the choice of priors and Markov chain Monte Carlo settings
☒	☐	For hierarchical and complex designs, identification of the appropriate level for tests and full reporting of outcomes
☐	☒	Estimates of effect sizes (e.g. Cohen's *d*, Pearson's *r*), indicating how they were calculated

Our web collection on statistics for biologists contains articles on many of the points above.

Software and code

Policy information about availability of computer code

Data collection	Neurophysiological data was acquired with a Plexon Omniplex system and and Plexon v-probes. The experiment was controlled by NIMH MonkeyLogic v2.0. Eye tracking was done with an Eyelink 1000 infrared tracker from SR Research. Recording grids were designed using Onshape and 3D printed using a Form Labs Form3+ printer.
Data analysis	Data were analyzed via custom scripts written in Matlab. The code is available at https://github.com/t-elston/cued_flex_val

For manuscripts utilizing custom algorithms or software that are central to the research but not yet described in published literature, software must be made available to editors and reviewers. We strongly encourage code deposition in a community repository (e.g. GitHub). See the Nature Portfolio guidelines for submitting code & software for further information.

Data

Policy information about availability of data

All manuscripts must include a data availability statement. This statement should provide the following information, where applicable:

- Accession codes, unique identifiers, or web links for publicly available datasets
- A description of any restrictions on data availability
- For clinical datasets or third party data, please ensure that the statement adheres to our policy

The data that support the findings of this study are available from the corresponding author upon reasonable request. Data are stored on controlled servers located at UC Berkeley.

Research involving human participants, their data, or biological material

Policy information about studies with human participants or human data. See also policy information about sex, gender (identity/presentation), and sexual orientation and race, ethnicity and racism.

Reporting on sex and gender	N/A
Reporting on race, ethnicity, or other socially relevant groupings	N/A
Population characteristics	N/A
Recruitment	N/A
Ethics oversight	N/A

Note that full information on the approval of the study protocol must also be provided in the manuscript.

Field-specific reporting

Please select the one below that is the best fit for your research. If you are not sure, read the appropriate sections before making your selection.

☒ Life sciences ☐ Behavioural & social sciences ☐ Ecological, evolutionary & environmental sciences

For a reference copy of the document with all sections, see nature.com/documents/nr-reporting-summary-flat.pdf

Life sciences study design

All studies must disclose on these points even when the disclosure is negative.

Sample size	Two non-human primates were used in this study. In accordance with the principle of "Reduce" in the 3 Rs of Responsible Animal Research framework, two NHPs is the standard sample size used in NHP neurophysiological research.
Data exclusions	No data were excluded.
Replication	Replication We analyzed the data from each subject separately and were able to successfully replicate the findings of the first monkey in the second one.
Randomization	No randomization was used as we did not perform an experiment which required it. However, the sequence of conditions within each behavioral/recording session was randomized.
Blinding	Blinding was not relevant to our study as our design did not involve a "treatment" or other manipulation that could be blinded.

Reporting for specific materials, systems and methods

We require information from authors about some types of materials, experimental systems and methods used in many studies. Here, indicate whether each material, system or method listed is relevant to your study. If you are not sure if a list item applies to your research, read the appropriate section before selecting a response.

Materials & experimental systems

n/a	Involved in the study	
☒	☐	Antibodies
☒	☐	Eukaryotic cell lines
☒	☐	Palaeontology and archaeology
☐	☒	Animals and other organisms
☒	☐	Clinical data
☒	☐	Dual use research of concern
☒	☐	Plants

Methods

n/a	Involved in the study	
☒	☐	ChIP-seq
☒	☐	Flow cytometry
☒	☐	MRI-based neuroimaging

April 2023

Animals and other research organisms

Policy information about studies involving animals; ARRIVE guidelines recommended for reporting animal research, and Sex and Gender in Research

Laboratory animals	Two male rhesus macaques (subjects D and K, respectively) aged 8 and 4 years, and weighing 11 and 9 kg at the time of recording were used in the current study.
Wild animals	This study did not involve wild animals.
Reporting on sex	Both of our subjects were male, therefore, we do not make any claims as to sex-related effects.
Field-collected samples	This study did not involve field-collected samples.
Ethics oversight	All procedures were carried out as specified in the National Research Council guidelines and approved by the Animal Care and Use Committee at the University of California, Berkeley.

Note that full information on the approval of the study protocol must also be provided in the manuscript.

Plants

Seed stocks	N/A
Novel plant genotypes	N/A
Authentication	N/A
